# Ontology extension by online clustering with large language model agents

**DOI:** 10.3389/fdata.2024.1463543

**Published:** 2024-10-07

**Authors:** Guanchen Wu, Chen Ling, Ilana Graetz, Liang Zhao

**Affiliations:** ^1^Department of Computer Science, Emory University, Atlanta, GA, United States; ^2^Rollins School of Public Health, Emory University, Atlanta, GA, United States

**Keywords:** ontology extension, online hierarchical clustering, large language model, medical ontology, zero-shot classification

## Abstract

An ontology is a structured framework that categorizes entities, concepts, and relationships within a domain to facilitate shared understanding, and it is important in computational linguistics and knowledge representation. In this paper, we propose a novel framework to automatically extend an existing ontology from streaming data in a zero-shot manner. Specifically, the zero-shot ontology extension framework uses online and hierarchical clustering to integrate new knowledge into existing ontologies without substantial annotated data or domain-specific expertise. Focusing on the medical field, this approach leverages Large Language Models (LLMs) for two key tasks: Symptom Typing and Symptom Taxonomy among breast and bladder cancer survivors. Symptom Typing involves identifying and classifying medical symptoms from unstructured online patient forum data, while Symptom Taxonomy organizes and integrates these symptoms into an existing ontology. The combined use of online and hierarchical clustering enables real-time and structured categorization and integration of symptoms. The dual-phase model employs multiple LLMs to ensure accurate classification and seamless integration of new symptoms with minimal human oversight. The paper details the framework's development, experiments, quantitative analyses, and data visualizations, demonstrating its effectiveness in enhancing medical ontologies and advancing knowledge-based systems in healthcare.

## 1 Introduction

In computational linguistics and knowledge representation, an ontology is a structured framework that organizes information by categorizing entities, concepts, and relationships within a specific domain to facilitate shared understanding. Ontology extension aims to enhance these structures by integrating new concepts, entities, and relationships, thereby improving their completeness, accuracy, and utility. Ontology extension enriches knowledge bases, enabling more nuanced data analysis, and is crucial in fields like biomedical research, where it integrates emerging discoveries into existing medical ontologies, enhancing disease diagnosis and treatment personalization. In artificial intelligence, ontology extension contributes to developing sophisticated natural language processing systems that understand and process human language with greater nuance and precision.

The evolving nature of knowledge domains presents challenges for ontology extension, including maintaining consistency, preserving knowledge integrity, and merging diverse information sources without ground truth data. This motivates the design of a zero-shot ontology extension approach. Existing methods (Memariani et al., 2021; Santosa et al., 2021; Behr et al., 2023) often rely on substantial annotated datasets or specific domain expertise, which are resource-intensive and may not transfer effectively to zero-shot scenarios. There is a need for a more flexible, data-independent model capable of accommodating new, unlabeled instances in a dynamic knowledge landscape without extensive retraining or expert intervention.

Zero-shot ontology extension faces two main challenges: (1) *Symptom Typingidentifying and classifying medical symptoms from unstructured online patient forum* data: This challenge entails the identification and classification of medical symptoms from unstructured online patient forum data. The complexity arises due to the nature of streaming and noisy data, which complicates the accurate detection and categorization of symptoms. (2) *Symptom Taxonomyhierarchically organizing and integrating symptoms into a pre-existing ontology*: This challenge involves the hierarchical organization and integration of symptoms into an existing ontology. The difficulty is multifaceted: First, it necessitates determining the appropriate upper branch within the hierarchical structure for the new symptom. Subsequently, it requires identifying whether there are semantically duplicated symptoms already present within the ontology. Finally, if the symptom does not align with any existing categories, it must be decided which category it should belong to, and this new relation must be added to the hierarchy. Thus, the challenges in zero-shot ontology extension are rooted in both the inherent characteristics of the data and the need for structured integration into a pre-existing ontological framework.

In this work, we propose a novel framework to address these challenges by employing LLM-powered agents and an online clustering system for efficient and effective Symptom Typing and Symptom Taxonomy. Specifically, given posts continuously from online health forums, we incorporate multiple LLM agents to identify and integrate new symptoms into existing ontology while considering the ontological hierarchy. The zero-shot framework can ensure accurate classification with minimal human oversight and without extensive labeled datasets.

This paper begins with an overview of related works, followed by the introduction of our proposed framework leveraging LLMs to address the challenges. We then present a series of experiments, including quantitative analysis and data visualization, to validate and demonstrate the efficacy of our framework. For our analysis, we used data from online patient forum discussion boards focused on breast and bladder cancer survivors.

## 2 Related work

Recent advancements in ontology extension methodologies have showcased diverse approaches to enriching and refining ontological structures. Deep learning techniques have been employed to automatically classify chemical structures within ontologies, significantly improving accuracy and providing insights into decision-making through attention-weight visualization (Memariani et al., 2021). Similarly, classification techniques have been used to automate the extension of computer science ontologies (Santosa et al., 2021). In the pharmacotherapeutic domain, semantic tagging and knowledge discovery methods from text corpora have been developed to automate ontology updates (Cruanes, 2011). For catalytic sciences, NLP-based concept extraction identifies and incorporates new categories related to catalytic reactions (Behr et al., 2023). Tools like Phrase2Onto leverage phrase-based topic modeling to facilitate ontology extension, proving effective in real-world applications and user studies (Pour et al., 2023).

Despite these advancements, these methodologies face limitations, including heavy reliance on domain-specific knowledge, extensive manual effort, and difficulties in adapting to the dynamic nature of knowledge. Additionally, integrating and validating new knowledge within existing structures and accurately capturing nuanced relationships without significant human oversight pose further challenges. These limitations highlight the need for more adaptive and sophisticated approaches in ontology extension that address both static and evolving aspects of knowledge domains.

In response, our research introduces an innovative multi-agent framework empowered by LLMs (Bai et al., 2024; Ling et al., 2023) that leverages zero/few-shot learning paradigms to bypass the extensive pre-training phase typical of conventional approaches. This framework enhances adaptability and extends applicability across a wider array of tasks, offering a versatile solution to ontology extension challenges. We rigorously scrutinize our proposed framework using a dataset derived from health-related forum posts.

## 3 Method

### 3.1 Problem formulation

In this paper, we addressed the problem of zero-shot ontology extension in the biomedical research domain, where new medical symptoms and their relations to one another constantly emerge. An ontology is a hierarchical structure with depth *k*, representing entities and their relationships within a domain to facilitate shared understanding. Our goal is to integrate novel symptoms into existing medical ontologies without requiring annotated data or substantial manual oversight. The problem is defined as follows:

Input:

- An existing ontology *O* with a hierarchical structure of depth *k*.
- Online streaming data *D*, containing domain specific texts.

Output:

- An extended ontology Õ, with new entities from *D* integrated into the existing hierarchical structure while maintaining ontological consistency.

### 3.2 Framework introduction

**Figure 1:**
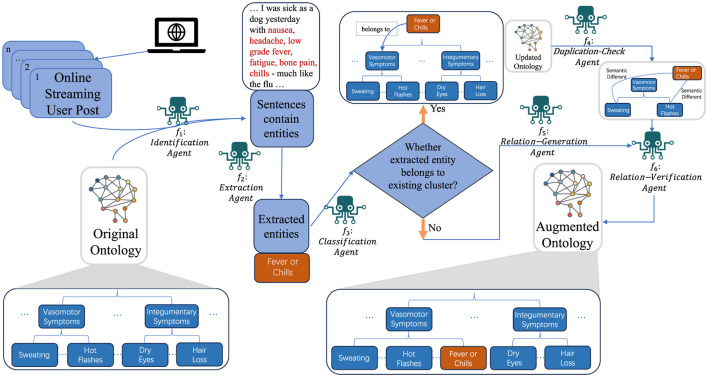
Overview of our proposed framework.

In this section, we introduced the detailed architecture of our proposed framework, as illustrated in Figure 1. Our study uses an existing hierarchical ontology and an online stream of unstructured textual data to generate an augmented hierarchical ontology. The initial step involves the precise identification and comprehensive summarization of entities within the streaming data. Entities refer to individual pieces of relevant information extracted from the text. Upon extraction, each entity is assigned to an appropriate cluster, which is a group of entities that share similar characteristics or are related in some meaningful way. For entities that align with pre-existing clusters, we check for semantic congruence to prevent duplication. For entities that do not fit into existing clusters, the framework dynamically creates new clusters to accommodate them.

#### 3.2.1 Challenges

While the problem may seem straightforward, existing methodologies fall short due to the following challenges: (1) handling continuously incoming data and (2) integrating multiple decision-making processes. Existing methods require meticulous fine-tuning and extensive model training, making them unsuitable for streaming data without prior dataset knowledge. The complexity of multi-dimensional ontology structures necessitates advanced decision-making capabilities beyond current methods. Additionally, the problem requires direct identification and summarization of entities from streaming data, with text summarization tasks needing a dedicated summarization model and identification tasks requiring a named entity recognition model. Existing methods struggle to integrate these tasks into a cohesive and effective process, highlighting the need for a more sophisticated intelligence system to manage these complex processes effectively.

### 3.3 Online hierarchical clustering

**Figure 2:**
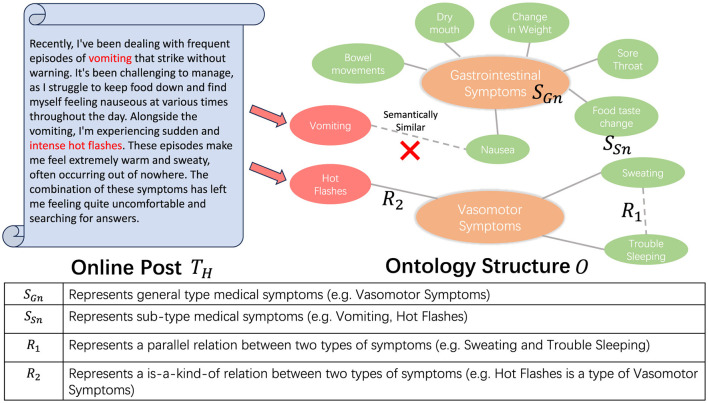
The figure presents a partial structure of an ontology, delineating the interrelations among various symptoms. For each symptom extracted, we categorize it into the appropriate pre-existing symptom groups (*S*_*Gn*_). In instances where the extracted symptom does not exhibit a *R*_2_ relationship with any *S*_*Gn*_, we earmark it for subsequent scrutiny.

Dealing with the complexities of streaming data requires us to process each piece of data as soon as it arrives. In this context, we conceptualize the ontology as an assemblage of clusters. Each cluster is represented by a centroid that typifies a general category of entities, with each constituent point representing a sub-type of these entities. In this work, we specifically target patient health forum post data, using medical symptom groups as clusters and medical symptoms as entities. In the following context, we will be using clusters to represent symptom groups and entities to represent medical symptoms. For example, as shown in Figure 2, the term “*Vasomotor Symptoms*” encompasses a group of interrelated symptoms, referred to as a cluster. Within this cluster, symptoms such as “*Hot Flashes*,” “*Sweating*,” and “*Trouble Sleeping*” are designated as entities. This approach enhances our ability to categorize and analyze data effectively.

Consequently, our task can be transformed into identifying the appropriate cluster for each incoming data. If each of the identified symptoms does not correspond to any pre-existing clusters, a new cluster will be created to encompass the identified entity. However, the assignment of an entity to a cluster is not a straightforward task. Upon determining the relevant cluster for the incoming data, it is essential to conduct a thorough analysis of semantic similarity within the cluster in order to avoid redundancy. To facilitate this, we propose a novel verification model, the specifics of which are discussed in Section 3.3.

Specifically, the input data for our algorithm includes an ontology *O* with a hierarchical depth of *k*, organized as hierarchical clusters, along with online streaming data *T*_*H*_. Additionally, the algorithm employs a set of sophisticated functions, *f*_1_ to *f*_6_, designed to process the streaming data effectively. The algorithm operates on the streaming data *T*_*H*_, examining each new sentence for relevant entities using function *f*_1_. When entities are detected (function *f*_2_), they are classified into existing clusters (function *f*_3_), which can handle the classification hierarchically based on the structure of the ontology *O*. For each identified entity *S*_*Sn*_, the algorithm determines whether it belongs to an existing cluster *S*_*Gn*_ or necessitates the creation of a new cluster $S_{Gn^{\prime }}$ (functions *f*_4_ and *f*_5_). Function *f*_4_ performs duplication checks and can also manage these checks hierarchically to ensure that entities are not redundantly assigned within the layered structure of *O*. Function *f*_5_ addresses the essential task of integrating symptoms that do not align with existing categories. It is responsible for creating new symptom clusters $S_{Gn^{\prime }}$ within the ontology. Utilizing a zero/few-shot learning methodology, this agent evaluates symptoms for potential parallel associations, thereby enabling the establishment of new categories that augment the ontology's breadth. This function can handle hierarchical relationships to ensure new clusters fit seamlessly into the existing layered structure. Finally, function *f*_6_ verifies the correctness of the entity-cluster assignment. This iterative process continues as long as new streaming data is available, thereby enabling dynamic and hierarchical clustering of entities based on the evolving data stream.

### 3.4 Multi-agent framework

In order to build a more sophisticated intelligence system to manage all the complex processes as summarized in Algorithm 1, we propose a novel multi-agent framework (Xi et al., 2023; Chen et al., 2024). In addition, in order to avoid extensive training on specific datasets, we strategically leverage LLMs as our executors to accomplish our task in a zero-shot manner. Specifically, we use LLM agents with specially designed prompts as the intelligent executors for functions *f*_1_ to *f*_6_.

**Algorithm 1:**
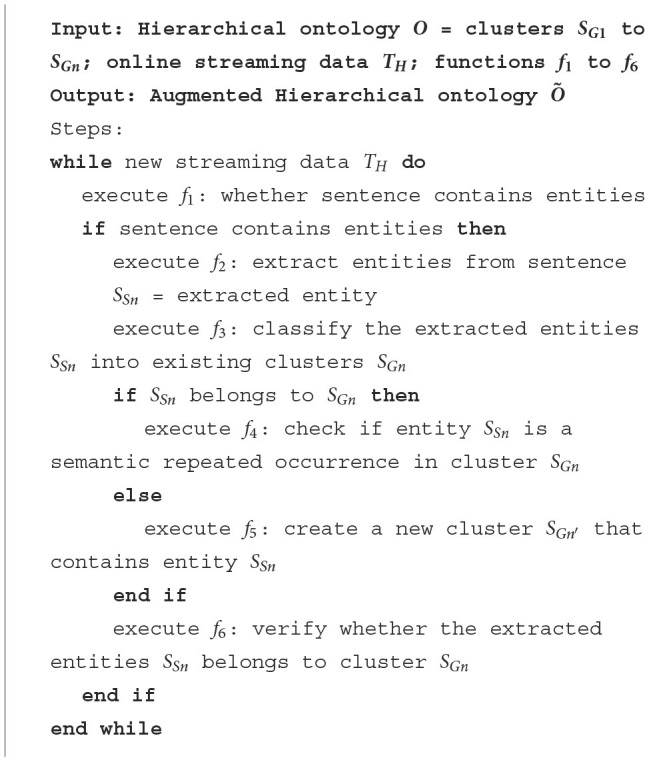
Online hierarchical clustering framework.

#### 3.4.1 *f*_1_: Identification agent

Initially, our endeavor involves the comprehensive parsing and analysis of the entirety of a given forum post. Through extensive empirical experimentation, we observed that the large language model exhibits heightened accuracy in identifying the presence of symptoms within individual sentences, as opposed to extracting symptom-containing sentences directly from entire forum posts. Consequently, we undertake a critical preprocessing step where the forum post data is meticulously segmented into individual sentences. The identification model is then tasked with thoroughly analyzing each sentence to determine whether it encompasses any symptoms, with all positive instances being systematically collated into a comprehensive list for further examination.

**Identification Agent Prompt**

**HumanMessage:** “Does the following sentence contain any symptoms? Sentence:” + sentence

**SystemMessage:** “Act as an experienced doctor. Given a sentence, if the sentence contains any symptoms, answer ‘yes', otherwise answer ‘no'.”

#### 3.4.2 *f*_2_: Extraction agent

The second large language model is then engaged, taking the symptom-containing sentences identified by the first model as its input. Its role is to extract the specific symptoms mentioned within these sentences and catalog them into a separate list for subsequent analysis.

**Extraction Agent Prompt**

**HumanMessage:** “Which words in the following sentence contain symptoms? Sentence:” + sentence

**SystemMessage:** “This is a set of symptom recognition problems. The ‘Sentence' is a sentence containing symptoms. The word ‘symptom' and treatments, like chemo, are not counted as symptoms. Also notice that human body parts, like knees and back, are not counted as symptoms. The goal is to return a Python list containing all possible symptoms, without any explainations. Here are some examples of what I want…”

#### 3.4.3 *f*_3_: Classification agent

Following the extraction phase, the Classification Agent engages in the systematic categorization of the symptoms into pre-existing clusters, as dictated by the ontology's established criteria. This process is facilitated through the deployment of a large language model to assess the alignment of each symptom with the pre-existing clusters. The agent handles things hierarchically by first determining the broad category of the symptom and then further refining its classification within subcategories of that broad category. Symptoms that fail to align with any of these clusters are segregated for subsequent analysis, ensuring the structured and accurate integration of symptoms within the ontological framework.

**Classification Agent Prompt**

**HumanMessage:** “Which existing category should the following string belongs to? String:” + sentence

**SystemMessage:** “This is a set of symptom classification problems. The ‘String' is a string describing symptoms. Use the following symptom_dictionary.json to answer the given questions. The structure of symptom_dictionary.json is: ‘existing category 1': ‘symptom 1', ‘symptom 2', ‘symptom 3', ‘existing category 2': ‘symptom 4', ‘symptom 5', ‘symptom 6'. The goal is to classify the string into one existing category. You only need to answer the name of the existing category should the string belongs. No explanation is needed. If the string does not belong to any existing category, simply answer ‘No'. Here are some examples of what I want…”

#### 3.4.4 *f*_4_: Duplication-check agent

The Duplication-Check Agent plays a crucial role in maintaining the integrity of the health symptom ontology by employing a zero/few-shot learning approach to prevent the inclusion of redundant symptoms. This method allows the agent to assess the uniqueness of symptoms without prior specific training on duplication cases. This agent possesses hierarchical capabilities by initially identifying duplicates at a broader categorical level, followed by a more detailed examination within finer subcategories. It relies on the intrinsic capabilities of language models to understand medical terminology and relationships, thus ensuring that only unique symptoms are added to the ontology. This hierarchical approach not only preserves the conciseness of the ontology but also enhances its utility for healthcare professionals by keeping the information distinct and valuable.

**Duplication-Check Agent Prompt**

**HumanMessage:** “Does this string a repeated occurrence in the exist_symptoms? String:” + sentence

**SystemMessage:** “This is a set of symptom classification problems. The ‘String' is a string describing symptoms. Use the following list of exist_symptoms to answer the given questions. exist_symptoms is a list that contains several strings describing symptoms. The goal is to determine if the string is a repeated occurrence in the exist_symptoms. You only need to answer 'no' if the string is not a repeated occurrence in the exist_symptoms and answer ‘yes' otherwise. No explanation is needed. + str(exist_symptoms) + Here are some examples of what I want…”

#### 3.4.5 *f*_5_: Relation-generation agent

Addressing the essential task of integrating symptoms that do not align with existing categories, the Relation-Generation Agent is tasked with the creation of new symptom clusters $S_{Gn^{\prime }}$ within the ontology. Utilizing a zero/few-shot learning methodology, this agent evaluates symptoms for potential parallel associations, thereby enabling the establishment of new categories that augment the ontology's breadth. This agent will assess the symptoms identified by the *f*_3_ agent, specifically focusing on those symptoms that fail to align with any existing clusters. If this agent identifies a symptom as belonging to a category that is not present in the original ontology, it subsequently integrates the new category-symptom relation into the ontology. The establishment of these new groups is instrumental in accommodating emerging medical knowledge and symptomatology within the ontology.

**Relation-Generation Agent Prompt**

**HumanMessage:** “Which broad category of symptoms does the given symptom belong to? Symptom:” + sentence

**SystemMessage:** “This is a set of symptom classification problems. Act as an experienced doctor. The ‘Symptom' is a string representing symptoms. Given a symptom, the goal is to classify the symptom into a general type of medical symptom. List the symptoms in the format of ‘XXX symptoms', such as Gastrointestinal symptoms, Gynecologic symptoms, and Musculoskeletal symptoms. No explanation is needed. Here are some examples of what I want…”

#### 3.4.6 *f*_6_: Relation-verification agent

The final phase of our comprehensive methodology is characterized by the Relation-Verification Agent, which employs an advanced LLM agent to perform a thorough review of the augmented ontology. This agent meticulously scrutinizes the classification and categorization of symptoms, ensuring their precise alignment with the correct *S*_*Gn*_. Through this rigorous verification process, the agent ensures the ontology's accuracy and relevance, thereby guaranteeing that the symptom categories reflect the most up-to-date medical insights and relationships.

**Relation-Verification Agent Prompt**

**HumanMessage:** “Does the string a symptom of the category? Category:” + category + “String:” + symptom

**SystemMessage:** “Act as an experienced doctor. Given a string, if the string belongs to the given category, answer ‘yes', otherwise answer ‘no'.”

## 4 Experiments

In this section, we will introduce the evaluation process of our proposed framework. First, we will introduce the dataset we used in the experiments, followed by the experiment process. Then we demonstrate the experimental results with a comprehensive discussion.

### 4.1 Dataset

#### 4.1.1 Forum posts

We used two cancer-related datasets from online forums. The first dataset focuses on breast cancer, comprising various medicines and related user posts. The second dataset pertains to bladder cancer, structured as a hash table with keys such as *datePublished, dateModified, author*, and *posts*, the last of which contains the text of user-generated posts. These distinct structures allowed us to effectively evaluate our framework's performance through user discussions on breast and bladder cancer. We segmented the forum posts into individual sentences, resulting in ~200,000 sentences for breast cancer and 76,000 for bladder cancer. For our experiments, we randomly selected 5,000 sentences from each dataset.

#### 4.1.2 Symptom table

We used an ontology to categorize symptoms discussed in the posts. This ontology is based on a study of breast cancer survivors, ensuring relevance and comprehensiveness in symptom categorization (Hu et al., 2022). The ontology includes categories such as Gastrointestinal symptoms (e.g., Change in Weight, Nausea), Gynecologic symptoms (e.g., Vaginal itching, Bleeding), Neuropsychologic symptoms (e.g., Headache, Dizziness), Vasomotor symptoms (e.g., Sweating, Hot flashes), Musculoskeletal symptoms (e.g., Joint pain, Muscle aches), Integumentary symptoms (e.g., Rash, Hair loss), Cardiorespiratory symptoms (e.g., Trouble breathing, Chest pain), Distress symptoms (e.g., Nervousness, Depression), and Despair symptoms (e.g., Feeling worthless, Hopelessness).

### 4.2 Implementation details

To design an effective and efficient framework, we used the OpenAI GPT-3.5-Turbo model for less computationally intensive tasks, such as the identification agent (*f*_1_). For more complex tasks, such as the relation-generation agent (*f*_5_), we used the OpenAI GPT-4-0125-preview model. This selection was made to enhance performance while optimizing API costs. We conducted quantitative analysis by masking around 60% of the symptoms in the original ontology and running the framework on 5,000 data entries from each dataset. The effectiveness of newly extracted symptoms in recovering the masked ones was assessed using a fuzzy score based on the Levenshtein Distance, with a score above 40 indicating successful recovery. Table 1 presents the experimental outcomes across both datasets, focusing on recall to determine the framework's ability to retrieve masked symptoms from the original ontology.

**Table 1:** Comparative analysis of symptom recovery performance in breast and bladder cancer forums using our proposed framework.

	**Breast cancer**			**Bladder cancer**		
	**PR**	**RE**	**F1**	**PR**	**RE**	**F1**
Gastrointestinal symptoms	35.7	100	52.6	16.1	83.3	27.0
Gynecologic symptoms	33.3	80.0	47.1	80.0	66.7	72.3
Neuropsychologic symptoms	38.5	100	55.6	27.2	60.0	37.5
Vasomotor symptoms	50.0	75.0	60.0	66.7	100	80
Musculoskeletal symptoms	11.3	100	20.3	63.6	100	77.8
Integumentary symptoms	41.2	100	58.3	41.7	71.4	52.6
Cardiorespiratory symptoms	62.5	100	76.9	75.0	75.0	75.0

The table displays Precision (PR), Recall (RE), and F1 Scores in percentage for different symptom categories. Symptom recovery was assessed after masking 60%–80% of symptoms in the original ontology, with the framework applied to 5,000 entries from each dataset. Recovery was determined by a fuzzy score greater than 40, based on the Levenshtein Distance, prioritizing recall to evaluate the ability to uncover the original ontology's symptoms.

Given the unique challenges of our problem, existing methods were not directly applicable for two primary reasons: (1) Existing methods are limited to pre-existing ontologies and clean, structured sentences, whereas our study involves online streaming data. (2) Extensive data cleaning and classification efforts are required in our research environment. To address these challenges, we developed two baseline models to evaluate the efficacy of our proposed framework. In the first baseline model (*separated approach*), we implemented natural language processing techniques to extract entities from streaming data. These entities, alongside the original ontology, were subsequently input into an LLM to generate an extended ontology. This method aims to leverage the LLM's advanced capabilities in ontology expansion while maintaining a clear delineation between entity extraction and ontology generation tasks. The second baseline model (*single-agent approach*) involves providing the LLM with a segment of the streaming data in conjunction with the original ontology to directly produce an extended ontology. This iterative process entails continuously supplying the LLM with new posts and the previously generated extended ontology until a comprehensive extended ontology is achieved. A critical constraint encountered in this approach is the prompt context window limit inherent to the LLM, which precludes the simultaneous input of the entire dataset. To navigate this limitation, we adopted an iterative approach that enables the LLM to incrementally process the data. This iterative methodology ensures that the model effectively synthesizes the streaming data and progressively refines the ontology within the confines of the context window limitations.

Additionally, we conducted an ablation study to analyze the contribution of different components within our framework. Specifically, we removed the Duplication-Check Agent (*f*_4_) and the Relation-Verification Agent (*f*_6_) and performed experiments on both datasets. This study assessed the impact of these agents on the overall performance and robustness of our framework.

### 4.3 Results

The experimental outcomes, as shown in Table 1, demonstrate the framework's performance across different symptom categories for breast and bladder cancer. Our primary focus is on the recall metric, as a higher recall score indicates the framework's capability to accurately recover masked symptoms. Lower precision scores in the table do not necessarily reflect diminished accuracy; instead, they may indicate a broader capture of symptoms that are not incorrect or irrelevant to the domain. An inspection of Table 1 reveals that half of the recall scores reached the maximum value of 100, indicating that the framework's generated augmented ontology accurately reconstructed the masked symptoms. The minimum recall score recorded is 60, showing substantial recovery for the remaining data. These results underscore the framework's efficacy in identifying and restoring symptom data, highlighting its potential utility in enhancing ontological structures.

We compared our proposed framework against two baseline models across both datasets, with the results presented in Tables 2, 3. In these tables, a hyphen indicates that the baseline output did not add any new symptoms to the respective symptom category, while a score of 0.00 indicates that the added symptoms had a fuzzy score of < 40. Table 2 shows that for the breast cancer dataset, the separated approach exhibited moderate performance with varying precision and recall scores across categories. For instance, Gastrointestinal symptoms had a precision of 50.0% and a recall of 28.6%, indicating some effectiveness in symptom recovery. However, certain categories, such as Gynecologic and Integumentary symptoms, had no additional symptoms generated. The single-agent approach showed improved recall in some categories, like Musculoskeletal symptoms (recall of 100), but struggled in others, reflected by zero precision and recall scores in several categories. Table 3 illustrates performance on the bladder cancer dataset, where the separated approach had variable results, with Gastrointestinal symptoms achieving a recall of 60.0% but a precision of 23.1%. The single-agent approach generally had low performance, with most categories showing zero or no new symptoms added, highlighting the challenge of generating relevant symptoms for this dataset. These baseline comparisons underscore the superior capability of our proposed framework in effectively recovering and categorizing medical symptoms, surpassing the baselines in most evaluated metrics.

**Table 2:** Results of the separated approach and single-agent approach on the breast cancer dataset, where 0.00 indicates that the augmented symptoms do not exhibit any association with the corresponding symptom category, and − indicates that the output did not add any new symptoms to the respective symptom category.

	**Separated approach**			**Single-agent approach**		
	**PR**	**RE**	**F1**	**PR**	**RE**	**F1**
Gastrointestinal symptoms	50.0	28.6	36.3	40.0	33.4	36.4
Gynecologic symptoms	0.00	0.00	0.00	–	–	–
Neuropsychologic symptoms	16.7	25.0	20.0	50.0	20.0	28.6
Vasomotor symptoms	50.0	33.3	40.0	50.0	33.3	40.0
Musculoskeletal symptoms	20.0	80.0	32.0	50.0	100	66.7
Integumentary symptoms	0.00	0.00	0.00	–	–	–
Cardiorespiratory symptoms	–	–	–	–	–	–

**Table 3:** Results of the separated approach and single-agent approach on bladder cancer dataset, where 0.00 indicates that the augmented symptoms do not exhibit any association with the corresponding symptom category, and − indicates that the output did not add any new symptoms to the respective symptom category.

	**Separated approach**			**Single-agent approach**		
	**PR**	**RE**	**F1**	**PR**	**RE**	**F1**
Gastrointestinal symptoms	23.1	60.0	33.3	20.0	60.0	30.0
Gynecologic symptoms	–	–	–	0.00	0.00	0.00
Neuropsychologic symptoms	16.7	25.0	20.0	0.00	0.00	0.00
Vasomotor symptoms	–	–	–	0.00	0.00	0.00
Musculoskeletal symptoms	20.0	80.0	32.0	0.00	0.00	0.00
Integumentary symptoms	–	–	–	0.00	0.00	0.00
Cardiorespiratory symptoms	–	–	–	0.00	0.00	0.00

Additionally, the results of our ablation study are presented in Table 4. While some symptom categories show higher recall scores compared to our proposed framework, there are notable declines in other metrics, particularly low precision scores and a marked decrease in F1 scores. The higher recall scores in certain categories can be attributed to the removal of Agent *f*_4_ and Agent *f*_6_, specifically the duplication-check agent and the relation-verification agent. The absence of these models in the ablation study prevented the framework from eliminating duplicate symptoms, thereby artificially inflating the recall scores relative to our proposed framework.

**Table 4:** This table presents the outcomes of an ablation study assessing the performance impact of omitting Agent *f*_4_ and Agent *f*_6_ from our proposed framework.

	**Breast cancer**						**Bladder cancer**					
	**Without agent** *f*_4_			**Without agent** *f*_6_			**Without agent** *f*_4_			**Without agent** *f*_6_		
	**PR**	**RE**	**F1**	**PR**	**RE**	**F1**	**PR**	**RE**	**F1**	**PR**	**RE**	**F1**
Gastrointestinal symptoms	17.2	100	29.4	20.0	100	33.3	14.6	100	25.5	17.1	100	29.3
Gynecologic symptoms	21.7	100	35.7	44.4	100	61.5	50.0	75.0	60.0	66.7	80.0	72.7
Neuropsychologic symptoms	6.06	100	11.4	9.30	100	17.0	17.4	100	29.6	20.0	75.0	31.6
Vasomotor symptoms	9.38	100	17.1	28.6	100	44.5	42.9	75.0	54.5	33.3	66.7	44.4
Musculoskeletal symptoms	3.31	100	6.41	6.06	100	11.43	27.3	100	42.9	25.0	100	40.0
Integumentary symptoms	10.7	100	19.4	12.2	100	21.8	38.9	100	56.0	30.8	80.0	44.4
Cardiorespiratory symptoms	12.9	100	22.9	23.5	100	38.1	28.6	100	44.5	50.0	66.7	57.1

Precision (PR), recall (RE), and F1 scores are reported for various symptom categories within datasets pertaining to breast and bladder cancer. The study's objective was to isolate and identify the contribution of specific models to the task of symptom classification and recovery, as measured by the established metrics of precision, recall, and F1.

In the data visualization segment of our study, we rendered three distinct graphs, each corresponding to one of the pre-existing symptom groups within the ontology. In these visual representations, blue nodes symbolize the symptoms initially contained in the ontology, while red nodes denote the symptoms introduced in the augmented ontology. We depicted the centroids of the clusters formed by the original symptom sets alongside those formed by the augmented symptoms and quantified the distance between these centroids. The minimal inter-centroid distances suggest a substantial semantic correlation between the symptoms incorporated into the augmented ontology and those pre-existing within the original ontology. This proximity implies that the additions to the ontology are semantically coherent, aligning closely with the established symptomatology.

### 4.4 Data visualization

**Figure 3:**
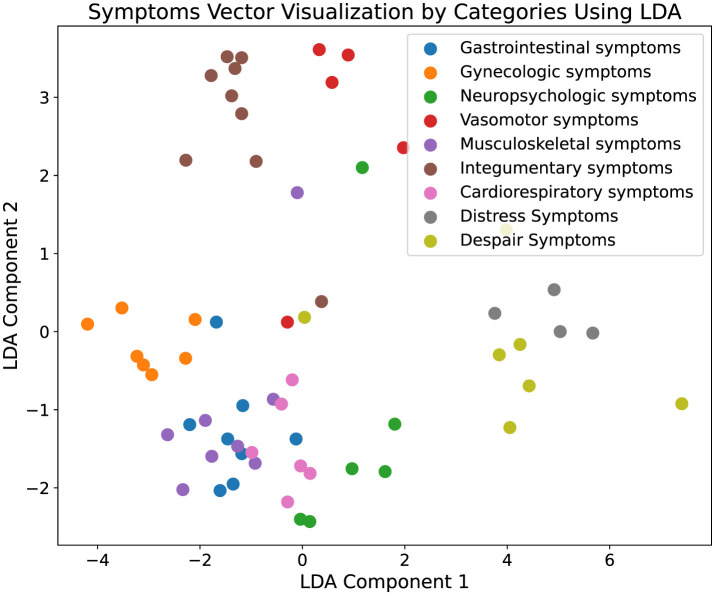
Visualization of the original ontology. This figure displays the results of a two-dimensional Latent Dirichlet Allocation (LDA) projection of symptom vectors categorized into various medical symptom types. Each point represents a symptom vector, and different colors denote distinct symptom categories, such as gastrointestinal symptoms and gynecologic symptoms. The visualization displays the clustering characteristics within each category, with points of the same color grouped closely together, indicating a high degree of semantic coherence. This effective clustering demonstrates the robustness of our framework in accurately categorizing and organizing complex medical symptoms into clearly defined groups, thereby validating the framework's effectiveness in enhancing the comprehensiveness and utility of medical ontologies.

**Figure 4:**
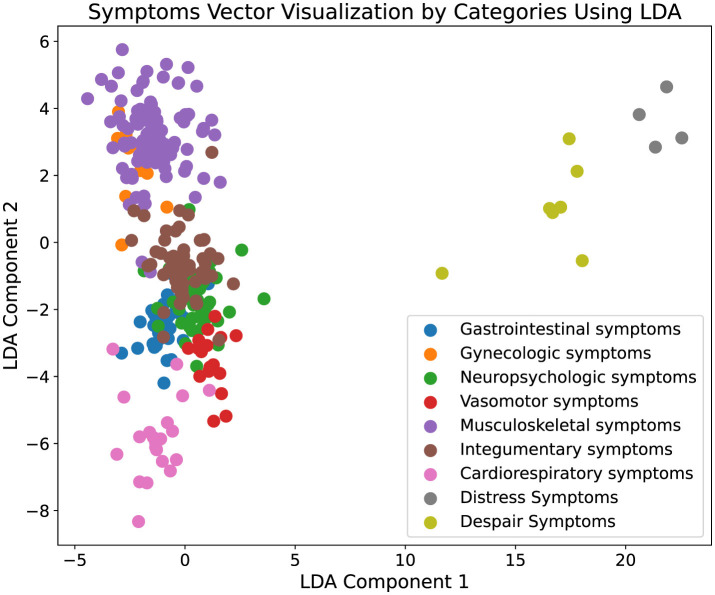
Visualization of the augmented ontology. This figure displays the results of a two-dimensional Latent Dirichlet Allocation (LDA) projection of symptom vectors categorized into various medical symptom types. Each point represents a symptom vector, and different colors denote distinct symptom categories, such as gastrointestinal symptoms and gynecologic symptoms. The visualization displays the clustering characteristics within each category, with points of the same color grouped closely together, indicating a high degree of semantic coherence. This effective clustering demonstrates the robustness of our framework in accurately categorizing and organizing complex medical symptoms into clearly defined groups, thereby validating the framework's effectiveness in enhancing the comprehensiveness and utility of medical ontologies.

In addition to quantitative analysis, our study incorporated data visualization to interpret the experimental results more intuitively. Initially, symptoms extracted from the augmented ontology were encoded using a BERT model to capture the high-dimensional semantic features of each symptom. These encoded features were then subjected to dimensionality reduction using Latent Dirichlet Allocation (LDA) to project the data into a two-dimensional space, facilitating a visual assessment of clustering characteristics. The resulting visualization, depicted in Figure 3, illustrates distinct clustering of symptom types, where each cluster is represented by a unique color corresponding to different symptom categories. The clear segregation of clusters indicates that symptoms within the same category are closely aligned in the reduced dimensional space, demonstrating spatial coherence and distinct separation from other categories. Moreover, Figure 4 presents the visualization results of the augmented ontology using LDA, further highlighting the clustering characteristics within each category. Points of the same color are tightly grouped together, indicating high semantic consistency. The distinct clustering observed in Figure 4 reinforces the reliability of our approach in maintaining semantic integrity and coherence across the augmented ontology, thereby validating the framework's capability in effectively extending and organizing medical symptom data.

## 5 Conclusion

In this paper, we explored the problem of zero-shot ontology extension and presented a comprehensive solution that incorporates unlabelled instances into existing ontologies. We leveraged LLMs to identify and integrate new entities and relationships, enhancing the structure and utility of ontological frameworks. Through experiments using patient discussion board data, we identified symptoms related to breast and bladder cancer and their treatments, demonstrating our framework's practical application. Our quantitative analysis and data visualization showed that our proposed framework improves the precision of entity classification and ensures that newly added entities maintain semantic coherence with the existing ontology. Furthermore, this approach has significant implications, including the potential to monitor in real-time the toxicity reported by patients for newly approved treatments, thereby improving patient safety and treatment efficacy.

However, it is important to note a limitation in our study. The original ontology we used in this paper is grounded in a comprehensive analysis of symptoms experienced by breast cancer survivors. This foundation ensures both relevance and thoroughness in symptom categorization. Consequently, it is important to acknowledge that many adverse symptoms pertinent to bladder cancer survivors, particularly those related to urinary issues, may not be adequately represented, as these issues are less common among breast cancer survivors. Being transparent about these limitations is crucial for the continued development and reliability of our ontology extension methodologies.

For future work, we aim to expand the framework's capabilities by incorporating multimodal data processing, allowing it to extend ontologies using diverse data types beyond text. For example, we plan to integrate medical imaging data and electronic health records (EHRs) to enhance the identification and classification of symptoms and treatments. Medical imaging data could be sourced from publicly available databases such as the Cancer Imaging Archive, while EHRs could be obtained from healthcare institutions with appropriate ethical approvals and data-sharing agreements.

Beyond the medical field, the framework also has potential applications in other domains, such as social networks. For instance, the framework could be used to automatically extend a social network ontology by analyzing new user data. Input data might include a user's profile features, activity patterns, and interactions, while the original social network ontology could contain categories like tags or groups based on interests, behavior, and preferences. The framework would then classify the new user into the most relevant category within the ontology. By leveraging zero-shot learning, the framework could identify and categorize emerging user behaviors or interests without requiring domain-specific training, showcasing its flexibility. We also plan to explore the framework's utility in areas like law and finance, where ontology extension could streamline complex document organization or enhance monitoring efforts. Through these advancements, we aim to establish our framework as a versatile tool in the evolving field of knowledge representation and engineering.

## Data Availability

The original contributions presented in the study are included in the article/supplementary material, further inquiries can be directed to the corresponding author.
